# Risk factors for mental health in general population during SARS-COV2 pandemic: a systematic review

**DOI:** 10.1186/s43045-022-00251-8

**Published:** 2022-10-11

**Authors:** Francesca Biondi, Marianna Liparoti, Angelica Lacetera, Pierpaolo Sorrentino, Roberta Minino

**Affiliations:** 1 Institute for Diagnosis and Care, Hermitage Capodimonte, Naples, Italy; 2 Department of Social and Developmental Psychology, University of Rome “Sapienza”, Rome, Italy; 3 Institut de Neuroscience Des Systemès, Aix-Marseille University, Marseille, France; 4 Department of Motor Sciences and Wellness, University of Naples “Parthenope”, Naples, Italy

## Abstract

The COVID-19 pandemic and its social restrictions have affected mental health globally. This systematic review aims to analyze the psychological responses of the general population and its related sociodemographic risk factors, excluding the most vulnerable groups (e.g., healthcare workers, COVID-19 patients and survivors, pregnant women, people with chronic diseases or preexisting psychiatric disorders). A reproducible search from June 2020 to February 2021 was conducted on PubMed and Google Scholar, following the PRISMA guidelines. Papers that (1) considered the most at-risk populations, (2) did not report sociodemographic data, and (3) did not use validated scales were excluded from our analysis. Non-English papers and review articles were also excluded. Of 1116 papers identified, 25 were included for this review (*n* = 162,465). The main risk factors associated with the emergence of depression, anxiety, sleep disorders, post-traumatic stress disorder, and obsessive compulsive disorder were: female gender, younger and later age, high level of education, Latino origin, free marital status, living quarantine in a house with no outdoor, negative coping strategies, close proximity to positive cases, high concern about contracting COVID-19 and living in a most affected area. High income, physical activity, resilience, family support, and a high level of knowledge about COVID-19, seems to be protective factors against the onset of psychological symptoms. In a general population, COVID-19 restrictions are linked to risk factors for psychological disorders caused by gender and sociodemographic conditions. In this regard governments should pay more attention to the public’s mental health and its risk and protective factors.

## Background

On March 11, 2020, the World Health Organization declared that the severe acute respiratory syndrome coronavirus (SARS-CoV2) outbreak in Wuhan, could be considered a global pandemic, named COVID-19 (coronavirus disease 2019) [1]. This new pandemic necessitated adapting immediately to a completely new reality, in which, for the good of all, it was essential to sacrifice some personal freedoms by isolating at home and maintaining social distance. The restrictions adopted by the governments of different countries, though they achieved a containment of infection in the first phase (World Health Organization, 2020), also affected people’s psychological well-being, causing emotional distress, anxiety, insomnia, depression, feeling of isolation, loneliness, boredom, and fear of being infected and infecting loved ones [2–7]. These negative reactions might have been increased by the growing number of new cases, the first deaths, and the extensive media coverage [8] that often deviates from medical and evidence-based sources, creating misinformation and alarmism [9].

Some population groups, e.g., healthcare workers, COVID-19 patients and survivors, pregnant women, people with chronic diseases or preexisting psychiatric disorder seem to be more vulnerable to the psychological effects of the pandemic [10, 11]. Most healthcare providers, exposed at the front lines in the fight against the virus [1], suffer from several disturbances such as depression (50.4%), anxiety (44.6%), insomnia (34%), and stress (71.5%) [12, 13]. In a study conducted in Wuhan by Zhu et al., the female health care workers or providers working with infected patients in emergency, intensive care or respiratory care, had increased risk of depression, anxiety, and stress [14]. They may have been particularly vulnerable due to close proximity to infected patients, long working hours, concerns about infecting loved ones, limited protective equipment, and involvement in emotional and ethical decisions [15, 16].

A large body of literature is available on the psychological outcomes in the most at-risk population; however, there are fewer studies about the psychological responses in the general population [6, 7, 17, 18]. The aim of this review is to analyze the role of sociodemographic variables in the appearance of psychological disorders in the general population during the pandemic. In particular, we analyzed the possible influence of sociodemographic variables, with special emphasis of gender (but not exclusively), on the onset of psychological disorders, such as depression, anxiety, sleep disorders, post-traumatic stress disorder (PTSD) stress and obsessive compulsive disorder (OCD) in the general population during the COVID-19 pandemic.

## Methods

Methods and results were developed using the Preferred Reporting Items for Systematic Reviews and Meta-Analyses (PRISMA) method [19]

### Search strategy

**Fig. 1:**
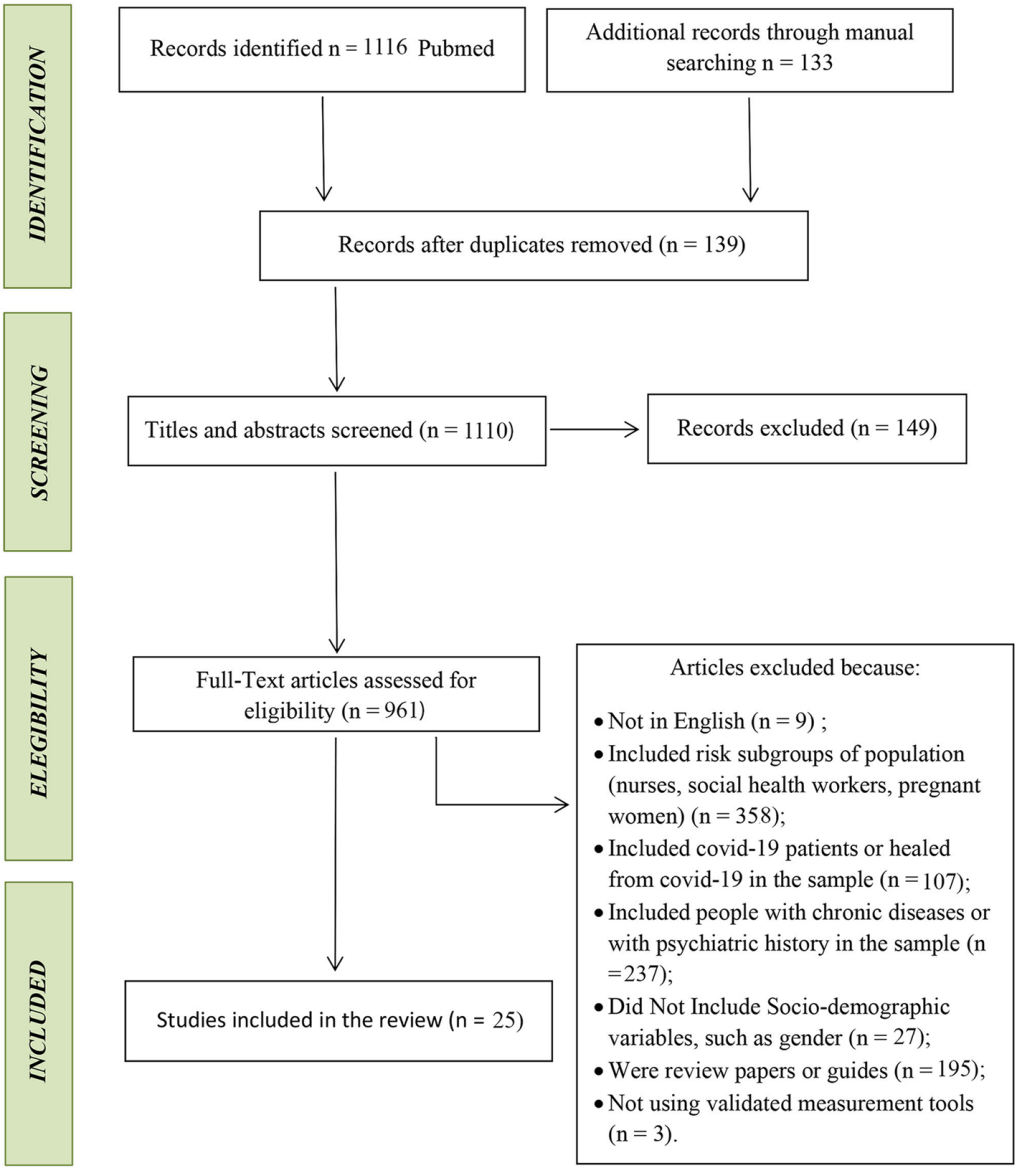
Adopted research methodology. The flow chart illustrates the steps of the selection procedures according to Preferred Reporting Items for Systematic Reviews and Meta-Analysis (PRISMA) study selection flow diagram

A systematic search using the PRISMA methodology was conducted from December 2020 to February 2021 on the PubMed platform. In the meantime, a manual search was performed on Google Scholar with the aim of identifying additional relevant studies (Fig. 1). The terms used were COVID-19 pandemic, mental health, psychological health, psychiatric disorders, depression, anxiety, sleep disorders, post-traumatic stress disorder, stress, obsessive compulsive disorder, gender-related, and social-demographic factors.

### Study selection and inclusion criteria

An initial selection was conducted by Titles and Abstracts. The second selection was made from full-text following the eligibility criteria. The inclusion criteria applied to the selected studies were (1) assessment of the mental health in the general population during the COVID-19 pandemic, (2) evaluation of related risk factors, (3) consideration of gender differences in the sample, and (4) outcomes using standardized and validated scales. Studies were not considered if they were not written in English, and if the sample examined included (1) COVID-19 patients or COVID-19 survivors, (2) subcategories of the population at high risk such as health care workers and pregnant women, and (3) subjects with histories of chronic illness or psychiatric diseases. Moreover, guides, reviews, and articles that did not contain sociodemographic data were not considered.

### Data extraction

In the preliminary analysis, the data extracted from the selected studies included (1) journal and author, (2) date of publication, (3) where the study was conducted, (4) study design, (5) period of administration, (6) sample size and characteristics, (7) disorders considered and diagnostic criteria, and (8) instruments/scales used.

Subsequently, a detailed analysis of the sociodemographic risk (or protective) factors related to the appearance of psychological disorders was carried out. More specifically, the risk and protective factors taken into account were (1) gender, (2) age, (3) level of education, (4) employment status, (5) ethnicity, (6) income, (7) region of origin, (8) marital status, (9) housing status, (10) relatives belonging to specific health categories, (11) frequency of physical exercise, (12) location of confinement/quarantine, (13) COVID-stressors (such as close contact with COVID patients, fear, concern about infection, knowledge of infected people, time spent researching COVID news, less knowledge about the virus, living in a high risk zone), and (14) psychological variables (i.e., coping strategy, resilience, tolerance of distress, social support).

## Results

### Research results

Globally, the identified publications were 1116, of which 139 were discarded as duplicates and 149 were excluded by screening titles and abstracts, leaving 961 full-text articles for eligibility. According to the exclusion criteria, 936 articles were discarded because they included (1) subgroups of the population at risk such as health care workers and pregnant women (n. 358); (2) COVID-19 or recovered patients (n. 107); (3) people with chronic diseases or histories of psychiatric illnesses (n. 237). Papers not in English (n. 9), articles with no sociodemographic information (n. 27), reviews, guides, or essays (n. 195) and articles with not validated assessment tools (n.3) were also excluded. After the selection process, 25 articles satisfied the inclusion criteria.

### Study characteristics

The main characteristics of the studies are summarized in Table 1. The number of subjects included in the studies ranged from 103 to 52,730, with a total of 162,465 participants (105,546 females, 56,762 males and 102 “others”). The range of the sample age varied from 6 to 60 years old. All works were cross-sectional studies, and the method used for sampling was “snowball sampling.” With regard to country, the studies were carried out in China (n. 10); the USA (n. 4); Italy (n. 2); Poland (n. 2); Greece (n. 1); Australia (n. 1); Lebanon (n. 1); Bangladesh (n. 1); Canada (n. 1), and Iran (n.1). Finally, one study was conducted worldwide (Australia, China, Ecuador, Iran, Italy, Norway, and the USA). Nine studies specifically discussed the psychological outcome of anxiety and depression, three the sleep disorders and three the correlation between anxiety, depression, and sleep disorders. Seven articles analyzed symptoms related to PTSD (among these articles, three also referred to symptoms related to depression, anxiety, and stress, and one also evaluated psychological distress). Three articles discussed the OCD symptoms.

**Table 1:** Summary of studies, sample characteristics, assessment tools, prevalence rates, and associated risk factors

**Lead author/year**	**Country**	**Sample size (*n*)**	**Sample characteristics**	**Assessment tool**	**Prevalence of the disorder (% or MeanScore)**	**Risk factors**
Zhou et al. 2020a [20]	China	8079	(range 12–18) M = 16 (years) Sex (f/m): 4326/3753	GAD-7 PHQ-9	Anxiety: 43.7% Depression: 37.4%	Gender, living area, education level
Chen et al. 2020 [21]	China	1036	Sex (f/m): 505/531	SCARED DSRS-C	Anxiety: 18.92% Depression: 11.78%	Gender, age group, education level
Islam et al. 2020 [22]	Bangladesh	476	Sex (f/m): 156/320	GAD-7 PHQ-9	Anxiety: 87.7% Depression: 82.4%	Gender, age, lagging academically, living area, housing status
Rudenstine et al. 2021 [23]	USA	1821	(range 18–77) M = 26.17 (years) Sex (f/m): 1301/ 493	GAD-7 PHQ-9	Anxiety: 41.3% Depression: 50.3%	Gender, age, education level, ethnicity, marital status, household income, COVID-19 stressors
Fawaz et al. 2021 [24]	Lebanon	520	(range 18–36) M = 21.03 ± 2.66) (years ± SD) Sex (f/m): 319/201	DASS‐21	Anxiety: 7.25(± 4.74) Depression: 7.67 (± 5.58)	Gender
Zhang et al. 2020c [25]	China	1018	M = 16.61 ± 1.06 (years ± SD)	GAD-7 PHQ-9	Anxiety: 31.4% Depression: 52.4%	Gender, education level
Hammarberg et al. 2020 [26]	Australia	13,762	Sex(f/m): 10,434/3328	GAD-7 PHQ-9	Anxiety: 20.0% Depression: 24.8%	Gender
Debowska et al. 2020 [27]	Poland	7228	M = 22.78 ± 4.40 (years ± SD) Sex (f/m): 5855/1373	DASS‐21	/	Gender
Fitzpatrick et al. 2020 [28]	USA	10,368	Age:18 and over Sex (f/m): 5290/5.078	CES‐D	Depression: 16.94	Gender, race, Hispanic origin
Bartoszek et al. 2020 [29]	Poland	471	(range 18–74) M = 25.5 ± 2.1 (years ± SD) Sex (f/m): 403/68	BDI ISI R-UCLA	Depression: 14.16 Insomnia: 15.58	Gender
Bigalke et al. 2020 [30]	USA	103	(range 18–68) M = 38 ± 1(years ± SD) Sex (f/m): 61/ 42	STAI PSQI ESS ISI CEDS	Anxiety 41 ± 1 Sleep disorders 7 ± 0 (psqi)/5 ± 0 (ess) Insomnia 7 ± 0 Depression 11 ± 1	Gender
Voitsidis et al. 2020 [31]	Greece	2427	(range 18–30) Sex (f/m): 1800/563	AIS	Insomnia: 37.6%	Gender, living area
Zhou et al. 2020b [32]	China	11,835	(range 12–29) Sex (f/m): 6826/5009	PSQI PHQ-9 GAD-7	Anxiety: 44.40% Depression: 44.80%	Gender, age, education level, living area
Wang et al. 2020 [6, 33]	China	6437	M = 31.40 ± 13.49 (years ± SD) Sex (f/m): 3613/2824	PSQI	Sleep disorders: 17.65%	Gender, age
Barrea et al. 2020 [34]	Italy	121	M = 44.9 ± 13.3 (years ± SD) Sex (f/m): 78/43	PSQI	Sleep disorders: pre 6.37 ± 3.96 post 8.64 ± 3.73	Gender
Qiu et al. 2020 [35]	China	52,730	(range18–60) Sex (f/m): 34,131/18599	COVID-19 PDI	Peritraumatic distress: 23.65 ± 15.45 Psychological distress: 35%	Gender, age, education level
Liang et al. 2020 [36]	China	570	(range14–35) Sex (f/m): 365/205	PCL-C GHQ-12 SCSQ	PTSD: 12.8%	Coping style
Liu et al. 2020 [18, 37]	USA	898	(range 18–30) Sex (f/m): 81.30%/14.1%	PCL-C GAD-7 PHQ-8	Depression: 43.3% Anxiety: 45.4% PTSD: 31.8%	Gender, age, loneliness, distress tolerance
Di Crosta et al. 2020 [38]	Italy	1253	(range18–64) Sex (f/m): 808/445	IES-R	PTSD: 35.59%	Gender, education level, COVID-19 stressors
Tang et al. 2020 [39]	China	2485	(range 16–27) Sex (f/m): 1525/960	PCL-C PHQ-9	PTSD: 2.7% Depression: 9.0%	Age, education level, COVID-19 stressors
Zhang et al. 2020b [25]	China	263	M = 37.7 ± 14 (years ± SD) Sex (f/m): 157/106	IES	Traumatic stress: 13.6 ± 7.7	No sociodemographic variable
Passavanti et al. 2021 [40]	Australia, China, Ecuador, Iran, Italy, Norway, USA	1612	M = 28 ± 9.36 (years ± SD) Sex (f/m): 968/644	IES-R PSS10 DASS-21 PHQ-9	PTSD: Iran M = 41.75, SE = 2.24; Perceived stress: Italy M = 21.14, SE = 0.76 Depression: Italy M = 18.49, SE = 1.23; Anxiety: Ecuador M = 13.39, SE = 1.36; Stress: Italy M = 20.60, SE = 1.32 Depression: Italy M = 10.44, SE = 0.78	Gender, type of housing, coping style, COVID-19 stressors
Abba-Aji et al. 2020 [41]	Canada	6041	Mage: 42 years Age range: 11–88 years Sex(f/m):5185/740	BOCS	Obsessions: 60.3% Compulsions: 53.8%	Gender, age, education level
Ji et al. 2020 [42]	China	Survay1: 13,478 Survay2: 8467 Survay3: 8816	Range: 17–50 years -Survey 1-sex (f/m):8816/4662 -Survay2-sex (f/m):5476/2991 -survay3-sex (f/m):5703/3113	Y-BOCS	Ocd: Survey1: 11.3% Survey2: 3.6% Survey3: 3.5%	Gender, age, COVID stressors
Darvishi et al. 2020 [43]	Iran	150	Range: 13–19 years Sex (f/m): 97/53	MOCI	OCD: 67.3%	Gender

Acronyms of questionnaires used: *AIS* = Athen Insomnia Scale [44], *BDI*** = **Beck’s Depression Inventory scale [45], *BOCS* = The Brief Obsessive–Compulsive Scale (Bejerotet al. 2014), *CES-D* = Center for Epidemiological Studies-Depression Scale [46], *COVID-19 PDI* = COVID-19 Peritraumatic Distress Index [35], D*ASS-21* = Depression, Anxiety, and Stress Scale-21 items [47], *ESS* = Epworth Sleepiness Scale (Johns MW, 1991), *GAD-7* = Generalized Anxiety Disorder 7 scale [48], *GHQ-12* = 12-Item General Health Questionnaire (Goldberg and Williams, 1988), *IES* = Impact of Event Scale [49], *IES-R* = Impact of Event Scale–Revised (Weiss and Marmar, 1997), *ISI* Insomnia Severity Index (Morin, Belleville, Bélanger and Ivers, 2011), *MOCI* = Maudsley Obsessive-Compulsive Inventory Questionnaire [50], *PCL-C* Post-traumatic stress disorder CheckList- Civilian Version ( Weathers, 1991); PH9-9 = Patient Health Questionnaire-9 [51], *PHQ-8* = Patient Health Questionnaire-8 (Kroenke et al. 2009), *PSQI* = Pittsburgh Sleep Quality Index [52], *PSS10* = Perceived Stress Scale 10 (Cohen, 1983), *R-UCLA* = Revised UCLA loneliness scale (Russell, D., Peplau, L.A., and Cutrona, C.E. 1980), *SCARED* = Screen for Child Anxiety-Related Disorders scale [53], *SCSQ* = Simplified Coping Style Questionnaire (Xie Y., 1998), *STAI* = State-Trait Anxiety Inventory scale (Spielberger, 1989), *Y-BOCS* = Yale-Brown Obsessive-Compulsive Scale (Goodman et al. 1989). Acronyms of mental disorders: *PTSD* = post-traumatic stress disorder

### Sociodemographic risk and protective factors

A variety of sociodemographic factors have been analyzed to identify risk or protective factors related to the appearance of the disorders were taken into account. The data collected are shown below.

#### Risk and protective factors for anxiety and depression

Depression is a very common disorder in the general population, in both physiological and specific pathological conditions, and results in physical and cognitive changes that affect human functioning [54, 55]. Depression is the most frequently identified disorder during the pandemic, often in comorbidity with other symptoms such as generalized anxiety or sleep disorders [56]. Specifically, depressive symptoms were evaluated in 9 out of the 25 studies. Three studies were conducted in China, [21, 25] (Zhou, Zhang, et al., 2020) two in the USA [23, 28], one in Poland [27], Australia [26], Bangladesh [22], and Lebanon [24]. Data were collected from March to May 2020. The depressive symptoms have also been evaluated in association with other psychological disorders, more specifically with: sleep disorders [29, 30] (Zhou, Wang, et al., 2020) and PTSD [37, 39, 40].

##### Gender

A strong association between gender and depressive symptoms has been observed by using both the Birleson Depression Self-Rating Scale for Children (DSRS-C) [21, 57] and the Center for Epidemiological Studies-Depression Scale (CES-D) [28, 46]. In particular, female gender is the main risk factor for the development of depressive symptoms [58]. Using the Patient Health Questionnaire-9 (PHQ-9 scale) [51], depressive symptoms were much less reported in males than in females [23, 25, 26, 40] (Zhou, Zhang, et al., 2020). By using other scales such as the Depression, Anxiety, and Stress Scale-21 items (DASS-21) [47], or the Beck’s Depression Inventory scale (BDI) [45], these results were also confirmed [27, 29, 30]. In contrast, Islam et al. observed higher prevalence of depressive symptoms in male students as compared to females [22]. Comorbid with anxiety symptoms was specifically evaluated in eight studies, three of which were conducted in China [21, 25] (Zhou, Zhang, et al., 2020) one in Bangladesh [22], USA [23], Lebanon [24], Australia [26], and Poland [27]. Data were collected from March 2020 to May 2020. Furthermore, in two studies, these symptoms were evaluated in association with sleep disorders. The most frequently used scale to quantify generalized anxiety was the Generalized Anxiety Disorder 7 scale (GAD-7) [48]. The results were highly consistent and showed the association of anxiety symptoms with female gender [23, 25, 26] (Zhou, Zhang, et al., 2020). However, Islam et al., although the same scale was used, reported a large prevalence of anxiety in male students [22]. The other scales used, such as the Screen for Child Anxiety-Related Disorders scale (SCARED) [53, 59] and DASS-21 [27] confirmed the role of female gender on the emergence of symptoms related to anxiety. The State-Trait Anxiety Inventory scale (STAI) [60] was used to assay both the State anxiety (which reflects transient anxiety) and the Trait anxiety (which assesses an individual’s predisposition to react with anxiety to any stressful event). By using this scale, the state anxiety of women showed higher values as compared to men. Finally, Passavanti et al., through the subscale Anxiety of DASS-21, did not observe any gender difference [40].

##### Age

Adolescents are one of the groups most at-risk. In fact, the pandemic has produced dramatic changes in their lifestyles. Younger age seems to be a risk factor for the development of symptoms of anxiety and depression. Chen et al. showed higher levels of anxiety (23.50%) and depression (21.15%) in the category from 13 to 15 years [21]. In Islam et al., the category from 21 to 24 years was the most affected by depression (66.07%) and anxiety (66.58%) [22]. Rudenstine et al. highlighted that the group between 18 and 39 years old showed higher levels of anxiety (43.0%) and depression (52.5%) [23]. Tang et al. [39] confirmed that the age range with the highest PHQ-9 scores was 18–30 [39].

##### Education

Educational level is an influencing factor in the development of anxiety and depression symptoms. According to Zhou et al., the prevalence of anxiety and depression was higher in the more educated population [13]. In Rudenstine et al., more severe symptoms of depression were recorded in the “high school diploma” category, and more severe symptoms of anxiety were recorded in the “college” category [23]. Tang et al. confirmed that a high degree of education is considered a risk factor for the emergence of depressive symptoms [39].

##### Ethnicity

Rudenstine et al. reported higher rates of anxiety and depression in people of Latino origin [23]. In Fitzpatrick et al., the population most affected by depressive symptoms was observed in the people of Hispanic origin [28].

##### Marital status

The Rudestine study showed higher levels of anxiety (42.6%) and depression (53.4%) in the category of “never married” [23], also confirmed by Fitzpatrick et al. [28].

##### Living area

Regarding living area, the data are inconsistent. According to Zhou et al. depression and anxiety seem to be more common in the people coming from rural areas (47.5% and 40.4%, respectively) as compared to that from urban area (37.7% and 32.5%, respectively) [25]. In contrast, Islam et al. described a higher prevalence in the sample coming from the urban area (depression 65.05%; anxiety 62.21%) [22].

##### Housing status

For the housing status variable, the results are also inconsistent. Islam et al. indicate that the subjects that live in the family showed significantly higher values of anxiety (96.40%), and depression (96.93%) than those that lives alone or away from the family [22], while according to Hammarberg et al., living in the family seems to be a protective factor [26].

##### Physical activity

The most important protective factor for the consequences of the outbreak was the physical exercise [61–64]. Indeed subjects that did not practice physical activity had higher rates of depression and anxiety compared to those that practiced regular physical activity during the pandemic period [21, 22].

##### Income

High income seems to be a protective factor regarding the onset of the depressive and/or anxiety symptoms [26]. This observation is also confirmed in Passavanti et al., which showed that higher level of depression was associated with low income [40]. Rudenstine et al. reported that belonging to a low–medium-income group increases the probability that depression and anxiety will arise [23].

##### COVID-stressors

High level of knowledge regarding COVID-19 prevention and control measures seems to be an important protective factor against the onset of symptoms related to anxiety and depression [25]. Close proximity to confirmed cases in the community [21], high levels of COVID-stressor presence [23], high concern about contracting the virus [26, 37], extreme fear, and infected acquaintances [39] were all considered risk factors closely related to the onset of anxiety and depression.

#### Risk and protective factors for sleep disorders

Sleep disorders were evaluated in six of the 25 studies included in this review. Two studies were conducted in China [7] (Zhou, Wang, et al., 2020), one in Poland [29], USA [30], Greece [31], and Italy [34]. Data were collected from February to May 2020.

##### Gender

Tang et al. showed that during the pandemic, sleeping less than 6 h a day is one of the risk factors closely related to the emergence of anxiety and depression [39]. Insomnia Severity Index (ISI) [65] scores were closely related to high scores of anxiety in women [30]. Voitsidis et al. [31], by using the Athen Insomnia Scale (AIS) [44] (the Greek version of the ISI scale) also underlined significantly higher scores in women. Similarly, Zhou et al. (Zhou, Wang, et al., 2020) and Barrea et al. [34] by using the Pittsburgh Sleep Quality Index (PSQI) [52], showed that the prevalence of insomnia symptoms was lower in males than in females. Wang et al. [33] using the same scale, also confirmed this result. Zhou et al. (Zhou, Wang, et al., 2020) using the PHQ-9 and GAD-7 scales to measure depression and anxiety respectively, observed that students who showed depressive or anxiety symptoms, also exhibited symptoms related to insomnia. Bigalke et al., by using the PSQI scale, reported that 66% of the population was classified as “poor sleepers,” but no correlation with the gender was found [30].

##### Age-related factor

In the Wang et al. study, participants over 50 years old showed higher values at the PSQI scale [33].

##### Housing area

For the housing area factor, the results are homogeneous. Voitsidis et al. and Zhou et al. collected data showing that living in urban areas was a risk factor for the appearance of symptoms related to insomnia, while living in the rural places was a protective factor [31] (Zhou, Wang, et al., 2020).

##### Physical activity

Physical exercise is also a protective factor for the onset of sleep disorders. In the study by Wang et al., the category that had maintained a frequency of sports activity of 3 or more times weekly during quarantine showed lower scores at PSQI scale [56], than those who exercised less or not at all [33].

##### COVID-stressors

High level of knowledge about COVID-19 has been proposed as a protective factor (Zhou, Wang, et al., 2020) for sleep disorders. Similarly, an optimistic outlook with the future vision about COVID-19 was associated with a lower rate of insomnia symptoms. In contrast, excessive worry about the current situation, specifically related to “perceived COVID-19 death” and “treatment difficulty,” was a risk factor for the onset of insomnia symptoms [33].

#### Risk and protective factors for post-traumatic stress disorder and stress

Post-traumatic stress disorder was seen in 7 out of the 25 studies. Four studies were conducted in China [35, 36, 39, 66], one in the USA [37] and Italy [38]. One research on PTSD and stress symptoms was conducted worldwide, including Australia, China, Ecuador, Iran, Italy, Norway, and the USA [40]. Data were collected in the period between January and May 2020.

##### Gender

The gender differences for PTSD’s symptoms were not homogeneous. In the research conducted by Zhang et al., the mean scores on the Impact of Event Scale (IES) [49] between males and females were not significantly different [66]. Also, in the post-traumatic stress disorder Check-List-Civilian Version (PCL-C) [67] scores, gender was not associated with PTSD, however gender moderated the direct effect between psychological distress and PTSD in males more than in females [36]. In the studies by Liu et al. [37] and Tang et al. [39], the scores at the PCL-C scale obtained by males and females were not different, but transgender male reported higher level of PTSD symptoms [37]. By contrast, in the studies of Di Crosta et al. and Passavanti et al., the Impact of Event Scale-revised (IES-R) [68] scores of females were higher than those of males [38, 40]. Women also showed higher levels of stress than males in the Perceived Stress Scale 10 (PSS-10) [69] scores and in the stress subscale of DASS-21 [40]. Also, in the COVID-19 Peritraumatic Distress Index (CPDI) scores, females showed significantly greater psychological distress than males [35].

##### Age

Both young and old age were risk factors for the development of PTSD, indeed 18–30 years old participants and those over 50 had higher probability of manifesting PTSD [35]. The young age as a risk factor was confirmed by both Tang et al. and Liu et al., who observed that the young participants reported more frequently PTSD symptoms [37, 39].

##### Education

High level of education was related to higher risk of developing distress in the CPDI, and it was also related to high scores in the PCL-C [39]. In contrast, in the research by Di Crosta et al., the less educated subjects exceed the cutoff on the IES-R scale [38].

##### Housing

The type of housing affected the level of perceived stress during the pandemic. In the study by Passavanti et al., participants who lived in a house with no outdoor space during quarantine had higher mean scores on the PSS-10 than those who lived in a house with a private garden [40].

##### Psychological variables

Negative coping strategies are significantly associated with PTSD [36]. Also, in the study of Passavanti et al., the avoiding coping strategy was associated with high scores in all scales [40]. In the study by Liu et al., 61.5% of participants reported feeling lonely during the pandemic [37]. In this study, loneliness and low distress tolerance appeared to be predictive factors for PTSD, whereas high levels of resilience, family support, and perceived instrumental support were related to low probability of developing PTSD.

##### COVID stressors

Living in an area of China most affected by COVID-19 was a risk factor for PTSD [35, 39]. However, according to Passavanti et al., Chinese participants exhibited lower levels of stress and PTSD compared to participants of other nationalities [40]. In particular, Italians showed the highest stress levels. A strong concern about infection, extreme fear of COVID-19, knowing infected people and seeking news about COVID-19 several times during the day, were all predictive factors for developing PTSD [37, 38, 39] and stress [40].

#### Risk and protective factors for obsessive compulsive disorder

Obsessive compulsive disorder symptoms were evaluated in 3 out of the 25 studies. One study was conducted in China [42], one in Canada [41], and one in Iran [43]. Data were collected from March to May 2020.

##### Gender

The study conducted by Darvishi et al. showed a prevalence of obsessive compulsive disorder’s symptoms in women (72.1%) than men (60.3%) [43]. Using the Maudsley Obsessional-Compulsive Inventory (MOCI) [50] underlined higher scores in women on the different subscales “Checking,” “Washing,” “Strictness,” and “Doubting.”

The studies conducted in China [42] and in Canada [41] showed that male gender had scores indicative of possible OCD. In the study of Abba-Aji et al., 63.2% of male participants are concerned about dirt and being infected with viruses and germs, compared with 60.1% of female participants [41]. Moreover, 57.3% of men wash their hands in a special way to avoid contaminations, compared with 53.4% of women.

##### Age

Younger and older age are risk factors for the development of OCD symptoms. In the study by Ji et al., the males under 26 years old show more symptoms than female younger than 26 years old [42]. In the study done in Iran by Darvishi et al., the mean age of onset of OCD among participants is 16.67 years [43]. In the research conducted by Abba-Aji et al., participants older than 60 years developed a greater concern about dirt, germs, and viruses and adopted more special hand washing, compared to younger age groups [41].

##### Education

High levels of education are a risk factor for the development of the OCD. 61% of participants with post-secondary education exhibit OCD symptoms, compared to 57.7% of participants with high school diploma and 52.8% of participants with lower levels of education [41].

##### Psychological variables

Participants who showed concern about dirt, viruses, and germs since the pandemic perceive increased stress, depressive symptoms [41], and symptoms of anxiety [41, 42].

##### COVID stressors

Fear intensity was positively related to OCD. Participants with possible OCD showed greater intensity of fear [42].

## Discussion

The restrictive measures adopted since the beginning of COVID-19 pandemic have undoubtedly slowed down the transmission of the virus (World Health Organization, 2020). However, the impositions of long periods of isolation, social distancing, and loss of personal freedoms have produced important psychological effects, causing the development of mental disorders and emotional distress [5]. These psychological outcomes have affected the population in different ways. Several groups proved more vulnerable, such as healthcare workers [1, 10], COVID-19 patients and survivors [70–73], chronically and mentally ill patients [74, 75], and pregnant women [11].

Research’s attention has been primarily directed to these risk groups, so in the literature few data are available on the general population [6, 7, 17, 37]. The objective of this review has been to investigate the impact of sociodemographic factors on the psychological responses to the pandemic in the general population. To achieve this goal, we considered only studies in the general population, excluding all studies involving the highest risk groups. The psychological disorders considered were depression, anxiety, sleep disorder, post-traumatic stress disorder, stress, and obsessive compulsive disorder.

The female gender seems to be the main sociodemographic risk factor for the development of disorders such as anxiety, depression, and insomnia. This result is in agreement with the existing literature, suggesting that women are twice as likely as men to develop symptoms of anxiety, depression [76–78] and sleep disorders [79]. Furthermore, depression is highly correlated with suicide attempts (72.4%) [80]. Dubè et al., in a meta-analysis of 54 studies, demonstrated an increase in suicidal ideation (10.81%) and suicide attempts (4.68%) during the COVID-19 pandemic, suggesting that female sex is a vulnerability factor for suicidal ideation [81]. From a social and cultural point of view, the pandemic has only highlighted existing cracks. Social norms and structures dictating that women assume caregiving roles are augmented during pandemics [82]. During this period because of the closure of schools, women have suffered a heavy psychological burden, not being able to rely on the help of grandparents in caring for their children due to social restrictions. Contrary to men, they often have to balance household burdens, responsibility over child rearing, and work pressures [83], still being relegated to the role of caregiver. Moreover, during the pandemic, the “glass ceiling” has hardened so the female unemployment rose to 11% compared to 7% in the males (Center for Law and Social Policy, 2020) and this could lead to further psychological distress.

Results on gender differences in PTSD symptoms are ambiguous. Three studies reported that women manifested more psychological symptoms [35, 38, 40], in line with a large part of the literature that associates women gender with a greater vulnerability to stress [84]. Women showed more reactivity than men in the fear processing that increases arousal response and risk of PTSD [85] However, in a study conducted in China [36], the effect of psychological distress on PTSD was significantly higher in males than in females. Chinese men are culturally seen as power figures, dominant especially in status and rights [59], but this socially acquired role might cause high psychological distress especially in this historical period because of the high economic instability. In the USA, men identified as transgender report high level of PTSD [37]. It is to note that COVID-19 pandemic has caused an increase in xenophobic attitudes toward minorities, such as LGBTQ people, because the novelty of illness and unknown’s fear might be associated with the presence of the “other” [86]. Concerning the gender difference in OCD, in two of the three studies [41, 42], OCD symptoms were highly observable in males compared to females, confirming data from the literature, according to which 70% of OCD patients are male [87, 88]. The predisposing factors of OCD are still unknown, but simply asking people to wash their hands, scrub, and sterilize in a ritualized and frequent manner, in order to protect themselves from the virus, may make people more anxious about their health and could lead to OCD occurring for the first time. This suggests that the combination of genetic factors, environment and psychological variables (e.g., fear, anxiety) could be a trigger factor in the etiology of OCD [42, 89].

Young age was an important risk factor for the emergence of mental disorder. Young people are much more exposed to media and social media, so to greater media impact, which could increase stress [35]. Losing relationships and other opportunities, young people are the ones who have sacrificed much to protect the elderly, who are most at risk of infection. Over 50 people are more likely to exhibit symptoms of mental disorders during pandemic because they are the most exposed to the risk of infection.

Interestingly, people with higher education levels show more symptoms of mental disorder than people with poor education. This may be because the pressures related to the pandemic are compounded with the elevated pressures associated with higher academic achievement, delays in studies and entry into the workforce [90].

High income is also a protective factor probably because it provides a sense of stability and security during periods of economic instability, such as this current period [23, 91]. Furthermore, during this pandemic, psychological and social resources become very relevant. Resilience, understood as the personal competence to withstand and adapt to adverse events, was associated with greater psychological well-being [37]. People’s ability to cope with stressful events has been important; in fact passive coping styles increase the risk of depression, anxiety, and PTSD symptoms [36, 40]. A passive approach tends to reinforce negative feelings, rejecting the existence of the stressful event in an attempt to avoid it. An active coping style is a protective factor for mental illness [92], because it enhances the individual’s ability to learn lesson in negative situation, to accept the existence of a negative situation, along with the ability to ask for help [93]. Social support, particularly family support, has a protective role for mental health during this period, because it has a stress buffering effect, improving the quality of life [69].

Finally, excessive worry and fear about the pandemic are risk factors for the emergence of psychological symptoms [6, 37, 39, 42]. Searching for COVID-19-related news several times throughout the day can increase vulnerability to exaggerated or even fake news, easily found especially on social media, increasing fear and worry [94]. Interestingly, some authors talk about “Headline Stress Disorder” caused by the bombardment of news related to COVID-19, causing physical symptoms such as insomnia and palpitations, and eventual mental disorders [95].

A possible limitation of the study is that the papers included in this review have a prevalence of female samples, making a gender-difference analysis not possible. Further studies assessing the effects of the pandemic on psychological health in men may be needed to investigate gender differences.

## Conclusions

This review is the first work to collect data on the influence of sociodemographic factors on the psychological responses to the pandemic in the general population, excluding at-risk groups (i.e., healthcare workers, pregnant women, chronically ill, mentally ill, COVID-19 patients, and survivors). This paper suggests that psychological disorders caused by restrictions during the pandemic depend on several sociodemographic risk factors. In fact, it seems that a higher incidence of the disorders highlighted in women than in men, younger and in people with and higher level of education. Conversely, it was observed that the sport practice, social and family supports, and higher income are protective factors. Therefore, a greater interest on the part of local and international governments in public mental health would be appropriate, in order to intervene and try to limit risks and improve the quality of life.

## Data Availability

Not applicable.

## Abbreviations

AIS: Athen Insomnia Scale
BDI: Beck’s Depression Inventory scale
CES-D: Center for Epidemiological Studies Scale
COVID-19: Coronavirus disease 2019
COVID-19 PDI: COVID-19 Peritraumatic Distress Index
DASS-21: Depression, Anxiety, and Stress Scale-21 items
DSRS-C: Depression Self-Rating Scale for Children
ESS: Epworth Sleepiness Scale
GAD-7: Generalized Anxiety Disorder 7 scale
GHQ-12: 12-Item General Health Questionnaire
IES: Impact of Event Scale
IES-R: Impact of Event Scale–Revised
ISI: Insomnia Severity Index
LGBTQ: Lesbian, gay, bisexual, transgender, and queer
MOCI: Maudsley Obsessional-Compulsive Inventory
OCD: Obsessive compulsive disorder
PCL-C: Post-traumatic stress disorder CheckList-Civilian Version
PH9-9: Patient Health Questionnaire-9
PHQ-8: Patient Health Questionnaire-8
PSQI: Pittsburgh Sleep Quality Index
PSS10: Perceived Stress Scale 10
R-UCLA: Revised UCLA loneliness scale
SCARED: Screen for Child Anxiety-Related Disorders scale
SCSQ: Simplified Coping Style Questionnaire
STAI: State-Trait Anxiety Inventory scale
PRISMA: Preferred Reporting Items for Systematic Reviews and Meta-Analyses
PTSD: Post-traumatic stress disorder
SARS-COV2: Severe Acute Respiratory Syndrome Coronavirus 2
UNICEF: United Nations International Children's Emergency Fund
